# HLA-Cw*0102-Restricted HIV-1 p24 Epitope Variants Can Modulate the Binding of the Inhibitory KIR2DL2 Receptor and Primary NK Cell Function

**DOI:** 10.1371/journal.ppat.1002805

**Published:** 2012-07-12

**Authors:** Lena Fadda, Christian Körner, Swati Kumar, Nienke H. van Teijlingen, Alicja Piechocka-Trocha, Mary Carrington, Marcus Altfeld

**Affiliations:** 1 Ragon Institute of MGH, MIT and Harvard, Charlestown, Massachusetts, United States of America; 2 Cancer and Inflammation Program, Laboratory of Experimental Immunology, SAIC-Frederick, Inc., Frederick National Laboratory for Cancer Research, Frederick, Maryland, United States of America; Emory University, United States of America

## Abstract

Accumulating evidence suggests an important role for Natural Killer (NK) cells in the control of HIV-1 infection. Recently, it was shown that NK cell-mediated immune pressure can result in the selection of HIV-1 escape mutations. A potential mechanism for this NK cell escape is the selection of HLA class I-presented HIV-1 epitopes that allow for the engagement of inhibitory killer cell immunoglobulin-like receptors (KIRs), notably KIR2DL2. We therefore investigated the consequences of sequence variations within HLA-Cw*0102-restricted epitopes on the interaction of HLA-Cw*0102 with KIR2DL2 using a large panel of overlapping HIV-1 p24 Gag peptides. 217 decameric peptides spanning the HIV-1 p24 Gag consensus sequence were screened for HLA-Cw*0102 stabilization by co-incubation with Cw*0102(+)/TAP-deficient T2 cells using a flow cytometry-based assay. KIR2DL2 binding was assessed using a KIR2DL2-IgG fusion construct. Function of KIR2DL2(+) NK cells was flow cytometrically analyzed by measuring degranulation of primary NK cells after co-incubation with peptide-pulsed T2 cells. We identified 11 peptides stabilizing HLA-Cw*0102 on the surface of T2 cells. However, only one peptide (p24 Gag_209–218_ AAEWDRLHPV) allowed for binding of KIR2DL2. Notably, functional analysis showed a significant inhibition of KIR2DL2(+) NK cells in the presence of p24 Gag_209–218_-pulsed T2 cells, while degranulation of KIR2DL2(−) NK cells was not affected. Moreover, we demonstrated that sequence variations in position 7 of this epitope observed frequently in naturally occurring HIV-1 sequences can modulate binding to KIR2DL2. Our results show that the majority of HIV-1 p24 Gag peptides stabilizing HLA-Cw*0102 do not allow for binding of KIR2DL2, but identified one HLA-Cw*0102-presented peptide (p24 Gag_209–218_) that was recognized by the inhibitory NK cell receptor KIR2DL2 leading to functional inhibition of KIR2DL2-expressing NK cells. Engagement of KIR2DL2 might protect virus-infected cells from NK cell-mediated lysis and selections of sequence polymorphisms that increase avidity to KIR2DL2 might provide a mechanism for HIV-1 to escape NK cell-mediated immune pressure.

## Author Summary

Distinguishing between “self” and “non-self” is one of the fundamental principles of immune responses against viral infections. Upon viral infection the peptide repertoire presented by HLA class I molecules changes, potentially providing signals that result in recognition and elimination of the infected cell by the host immune system. Viruses, in particular HIV-1, developed multiple strategies to escape T cell and Natural Killer (NK) cell-mediated immune pressure, including sequence variations that lead to the engagement of inhibitory receptors expressed on T cells and NK cells. The systematic approach used in this study led to the identification of an HLA-presented HIV-1 peptide that allows engagement of the inhibitory NK cell receptor KIR2DL2 and inhibition of NK function. Our findings help to elucidate the complex interaction between KIR molecules, such as KIR2DL2, and HLA/peptide complexes and provide a foundation for further studies investigating the role of sequence variations within HIV-1 epitopes on HLA/KIR interactions, and the ability of viruses to evade NK cell-mediated recognition.

## Introduction

Natural Killer (NK) cells play a pivotal role in the first line innate immune response to viral infections through the killing of virus-infected cells and modulation of adaptive immunity [1]–[3]. Their function is controlled by a number of surface receptors, including activating and inhibitory killer cell immunoglobulin-like receptors (KIRs) that interact with human leukocyte antigen (HLA) class I molecules expressed on target cells [4]. An increasing number of epidemiological and functional studies also suggest that NK cells might participate in the control and outcome of HIV-1 disease [5], [6]. While it was known that expression of *HLA-B* alleles with the serological Bw4 motif (HLA-Bw4) is protective in HIV-1 infection [7], [8], Martin *et al.* first demonstrated that the combined presence of alleles encoding for the activating receptor *KIR3DS1* and *HLA-B Bw4-80Ile* was associated with delayed progression to AIDS [9]. These data were supported by subsequent studies demonstrating enhanced function of KIR3DS1-expressing NK cells in *HLA-B Bw4-80Ile*(+) individuals and efficient *in vitro* suppression of HIV-1 replication in HLA-B Bw4-80Ile(+) target cells by KIR3DS1(+) NK cells [10], [11]. Better control of HIV-1 viremia was also described in individuals encoding for *HLA-B Bw4*alleles and certain subtypes of the highly polymorphic inhibitory receptor *KIR3DL1* linked to high expression of KIR3DL1on the surface of NK cells [12]. Interestingly, both KIR3DS1(+) and KIR3DL1(+) NK cells are preferentially expanded in acute and chronic HIV-1 infection, respectively, but only in individuals also encoding for the HLA-Bw4 ligand family [13]. More recently, it has also been described that HIV-1 selects for viral sequence polymorphisms associated with the expression of specific KIRs on the population level, suggesting escape from KIR-positive NK cell-mediated immune pressure [14]. Taken together, these data indicate that the interaction between KIR and HLA class I molecules plays a critical role in the control of HIV-1 infection. However, the underlying mechanisms by which NK cells recognize HIV-1-infected cells and how KIR/HLA interactions are involved in this context are not understood.

The recent description of the crystal structure of several KIR/HLA class I complexes has started to provide some insights into the rules that determine these interactions [15]–[17]. While the specificity of KIRs to interact with their cognate HLA class I molecules is defined by motifs located in the heavy chain of HLA, binding of KIR to HLA class I ligands is also affected by the HLA-bound peptides [18]–[22]. In particular, residues 7 and 8 of the HLA class I-presented peptide seem to be crucial in either promoting or compromising the binding affinity of KIRs [15], [23]–[28]. Therefore, alterations of the HLA-presented peptide repertoire occurring during viral infections might modulate binding of activating and inhibitory KIRs, and influence subsequent activation of NK cells [29]–[31]. Here we used a large panel of overlapping HIV-1 p24 Gag peptides to assess the impact of sequence variations of HLA-Cw*0102-presented HIV-1 epitopes on the binding of the inhibitory receptor KIR2DL2 and their consequences on primary NK cell function.

## Results

### Several HIV-1 p24 Gag peptides stabilize HLA-Cw*0102-expression on T2 cells

**Figure 1:**
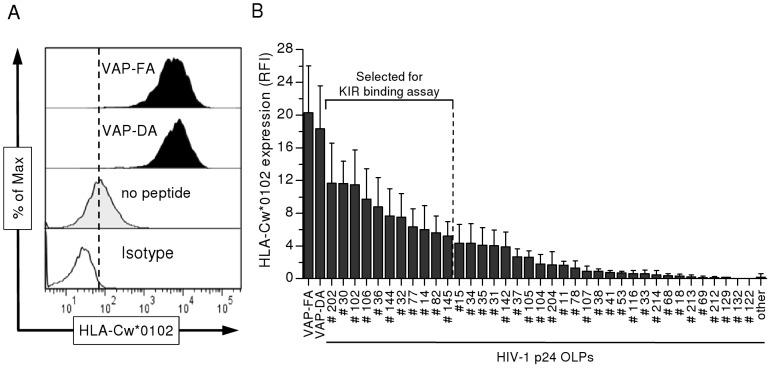
HIV-1 p24 peptide-dependent HLA-Cw*0102 stabilization on T2 cells. (**A**) Representative histograms of HLA-Cw*0102 stabilization on T2 cells as determined by flow cytometry. Histograms display HLA-Cw*0102 expression on T2 cells in the presence of control peptides VAP-FA and VAP-DA (black) at a concentration of 40 µg/ml as compared to unloaded T2 cells (grey tinted) and isotype control (clear). (**B**) HLA-Cw*0102 expression on HIV-1 p24 peptide-pulsed T2 cells. HLA-Cw*0102 expression is illustrated as relative median fluorescence intensity (RFI) as compared to unloaded T2 cells. Each column represents mean±SEM RFI of 5 independent experiments for each HIV-1 p24 OLP. A total of 59 HIV-1 p24 OLPs were analyzed, which previously showed strongest HLA-A/B/C stabilization as determined by an HLA-A/B/C antibody. Of those, 11 peptides with the highest HLA-Cw*0102 expression (>five-fold) were selected for subsequent KIR-Fc binding assays.

The goal of this study was to investigate the effect of HLA-Cw*0102-presented HIV-1 p24 Gag epitopes on binding to the inhibitory receptor KIR2DL2, and the impact on NK cell function. We initially identified HLA-Cw*0102-stabilizing epitopes in the HIV-1 p24 Gag protein. A total of 217 decameric peptides, overlapping by 9 amino acids (aa), were initially screened for general HLA class I stabilization using a pan HLA-A/B/C antibody by co-incubation with the TAP-deficient T2 cell line, and we subsequently screened HLA class I-stabilizing peptides for Cw*0102 stabilization using the HLA-C-specific antibody DT9. As positive controls, two previously described HLA-Cw*0102-restricted peptides [VAPWNSDAL (VAP-DA) and VAPWNSFAL (VAP-FA)] were used for monitoring HLA-Cw*0102 expression on the surface of T2 cells (Figure S1A and 1A) [23]. We identified several overlapping p24 Gag peptides (OLPs) which were associated with increased expression of HLA class I as determined by the HLA-A/B/C-specific antibody W6/32 (Table S1). Marked upregulation of HLA class I expression by more than 30% compared to un-pulsed T2 cells was observed for 59 different p24 OLPs (gray shading). Notably, the selected OLPs were not distributed randomly throughout the p24 Gag protein but rather clustered in certain regions. Confirmation of HLA-Cw*0102-specific stabilization for the 59 peptides that increased HLA class I expression was performed by using the HLA-C-specific antibody DT9 (Figure 1A). While T2 cells loaded with the previously described HLA-Cw*0102 epitopes VAP-FA and VAP-DA displayed high expression of HLA-Cw*0102, the majority of the 59 selected OLPs did not stabilize HLA-Cw*0102 expression on the surface of T2 cells (Figure 1B), suggesting presentation by either one of the other HLA class I molecules expressed on T2 cells, i.e. HLA-A*0201 or HLA-B*51 [32], [33]. However, 11 OLPs within p24 Gag were identified that induced a more than five-fold increase of HLA-Cw*0102 expression as compared to non-peptide pulsed T2 cells. All of these 11 OLPs contained binding motifs for HLA-Cw*0102, with 10/11 being ranked in the top 25% of HLA-Cw*0102 binders using either the NetMHC or the IEDB online prediction tool (Table S2). In sum, we identified 11 peptides within HIV-1 p24 Gag that were selected for subsequent studies assessing the consequences of these HLA-Cw*0102-presented epitopes for KIR binding.

### A single HLA-Cw*0102-restricted HIV-1 p24 epitope identified enables binding to KIR2DL2 and results in functional inhibition of primary NK cells

**Figure 2:**
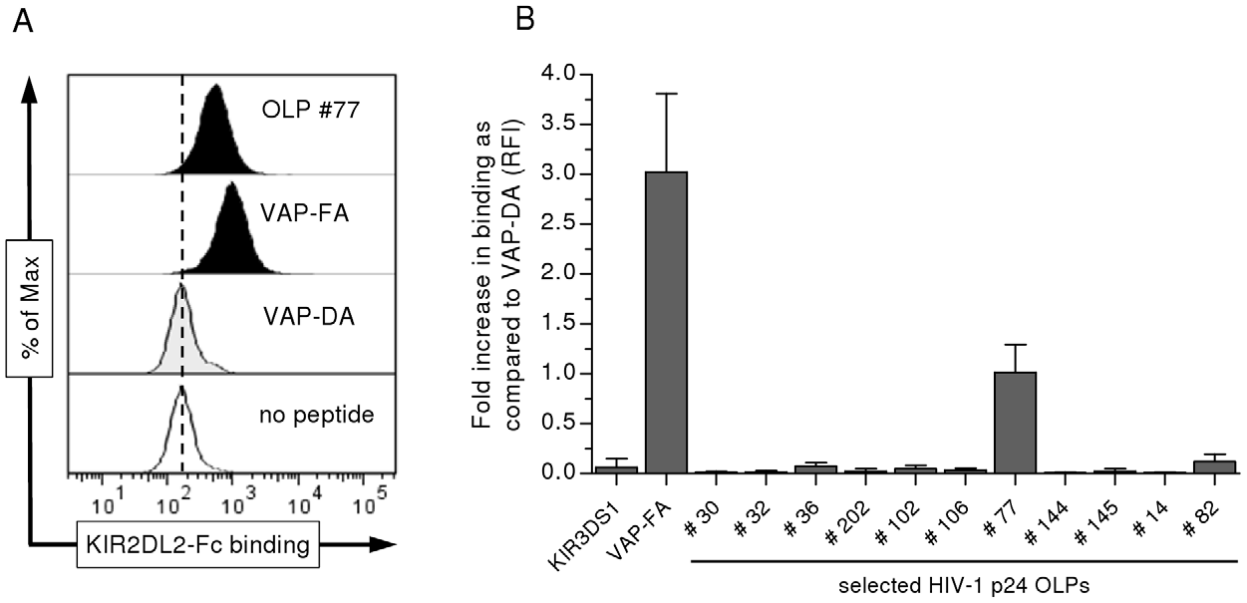
Differential binding of KIR2DL2-Fc to HIV-1 p24 peptide/HLA-Cw*0102 complexes. (**A**) Representative histograms of binding of KIR-Fc to T2 cells after peptide-induced HLA-Cw*0102 stabilization as determined by flow cytometry. From the top, histograms display binding of KIR2DL2-Fc in the presence of peptide #77 (p24 peptide; filled),VAP-FA (positive control, filled), VAP-DA (negative control, tinted), all at a concentration of 100 µM/ml, as compared to unloaded T2 cells (clear).(**B**) Relative binding of KIR2DL2-Fc to selected HIV-1 p24 peptide-pulsed T2 cells. KIR2DL2-Fc binding is illustrated as relative median fluorescence intensity (RFI) as compared to VAP-DA-pulsed T2 cells. KIR3DS1-Fc was used as a negative control to exclude unspecific binding of KIR-Fc molecules to VAP-FA-loaded T2 cells. Each column represents mean±SEM RFI of 5 independent experiments for each HIV-1 p24 OLP.

To determine the consequences of these HLA-Cw*0102-presented epitopes for KIR2DL2 binding, T2 cells were loaded with the selected peptides and then stained with a KIR2DL2-IgG fusion construct. As previously described by Fadda *et al.* VAP-FA and VAP-DA-loaded T2 cells served as positive and negative controls for the assessment of KIR2DL2-Fc binding (Figure 2A) [23]. In addition, a KIR3DS1-Fc was used as a negative control to exclude unspecific binding of KIR-Fc molecules to VAP-FA-loaded T2 cells (Figure 2B). The results of repeated KIR2DL2 binding assays are summarized in Figure 2B. As expected, we observed that VAP-FA-loaded T2 cells showed significant binding to KIR2DL2-Fc (>three-fold as compared to VAP-DA), while VAP-DA did not allow for KIR2DL2-Fc binding, despite stabilization of HLA-Cw*0102 expression. Most of the tested HLA-Cw*0102-presented HIV-1 p24 OLPs caused no or only very little binding (<0.2-fold increase as compared to VAP-DA) of HLA-Cw*0102 to KIR2DL2-Fc, with the exception of p24 OLP #77 (AAEWDRLHPV, referred to asp24 Gag_209–218_). In complex with HLA-Cw*0102, p24 Gag_209–218_ led to considerable binding of KIR2DL2-Fcto HLA-Cw*0102(>100% increase as compared to VAP-DA). To further determine the consequences of different peptide concentrations on HLA-Cw*0102-stabilization and KIR2DL2-Fc binding, we performed subsequent titration experiments (Figure S2). With increasing concentrations of peptide or KIR2DL2-Fc, HLA-Cw*0102-stabilization and KIR2DL2-Fc binding reached a level of saturation demonstrating that both, HLA-Cw*0102 stabilization and KIR2DL2-Fc binding were concentration-dependent. Taken together, only one out of 11 HLA-Cw*0102-stabilizing p24 Gag peptides allowed for the binding of the inhibitory receptor KIR2DL2.

**Figure 3:**
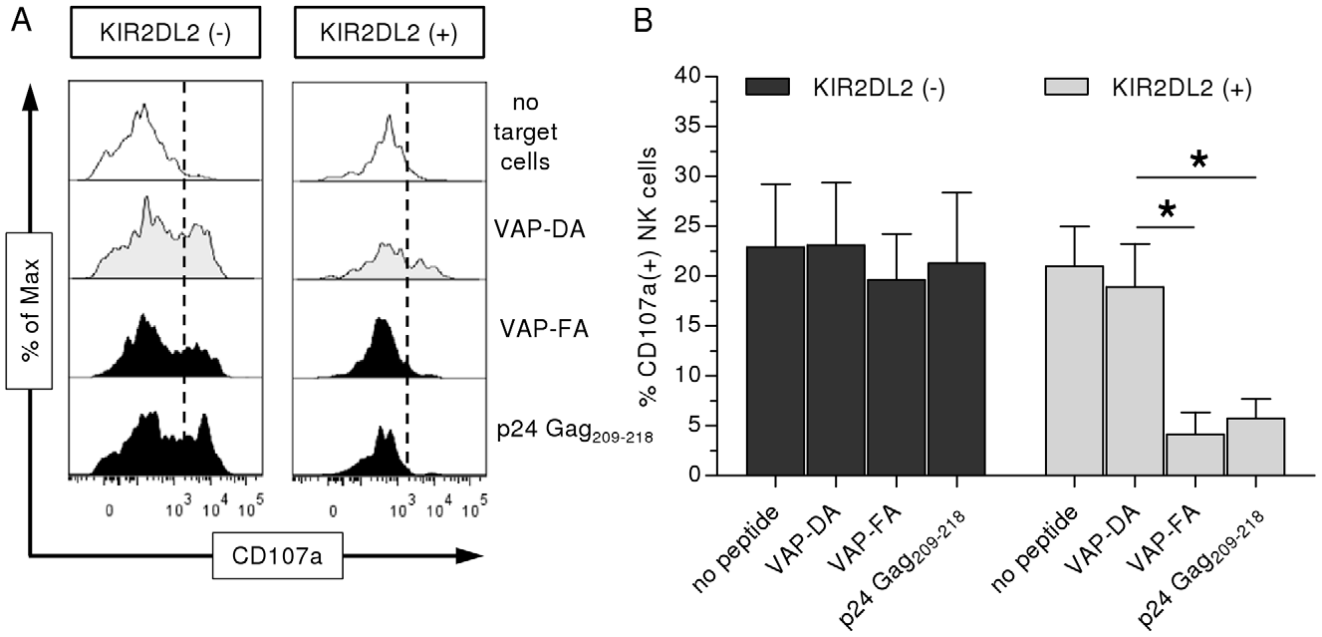
p24 Gag_209–218_/HLA-Cw*0102 complexes on T2 cells lead to functional inhibition of primary KIR2DL2(+) NK cells. (**A**) Representative histograms of degranulation of KIR2DL2(−) (left panel) and KIR2DL2(+) (right panel) NK cells after co-incubation with peptide-pulsed T2 cells (tinted and black) or without target cells (clear). NK cell degranulation was measured flow cytometrically by surface expression of CD107a. (**B**) Different levels of NK cell degranulation between KIR2DL2(−) (dark grey) and KIR2DL2(+) (light grey) NK cells after co-incubation with peptide-pulsed T2 cells. Each column represents mean±SEM percentage of CD107a(+) NK cells of 5 of different individuals. Unspecific degranulation of NK cells measured without target cells was deducted from each column.

We subsequently investigated whether binding of KIR2DL2 to p24 Gag_209–218_/HLA-Cw*0102 complexes *in vitro* affected effector functions of NK cells. Primary NK cells derived from *KIR2DL2(+)* individuals (Table S3) were co-incubated with T2 cells in the presence or absence of p24 Gag_209–218_, or control peptides, as indicated. As displayed in Figure 3A, in the absence of peptides or the presence of the VAP-DA peptide that did not allow for KIR2DL2 binding, T2 cells induced strong NK cell degranulation measured by the proportion of CD107a(+) NK cells. In contrast to the VAP-DA peptide, the two peptides VAP-FA and p24 Gag_209–218_ that allowed binding of KIR2DL2-Fc caused significant inhibition of degranulation of KIR2DL2(+) NK cells in response to peptide-pulsed T2 cells, while expression of CD107a on KIR2DL2(−) NK cells was not affected by the presence of these peptides (Figure 3B). In comparison to co-incubation with VAP-DA-pulsed T2 cells, the proportion of CD107(+) KIR2DL2(+) NK cells was reduced by 81±10% in the presence of VAP-FA-loaded T2 cells and 71.6±6.4% in the presence of p24 Gag_209–218_-pulsed T2 cells respectively (each p<0.05). Altogether, these functional data show that the HLA-Cw*0102-restricted p24 Gag_209–218_ peptide that enabled KIR2DL2 binding also resulted in the inhibition of primary KIR2DL2(+) NK cells, but not KIR2DL2(−) NK cells from the same individuals.

### Binding of KIR2DL2 to HLA-Cw*0102/peptide complexes is strongly effected by sequence variations in position 7 of the p24 Gag_209–218_ peptide

**Figure 4:**
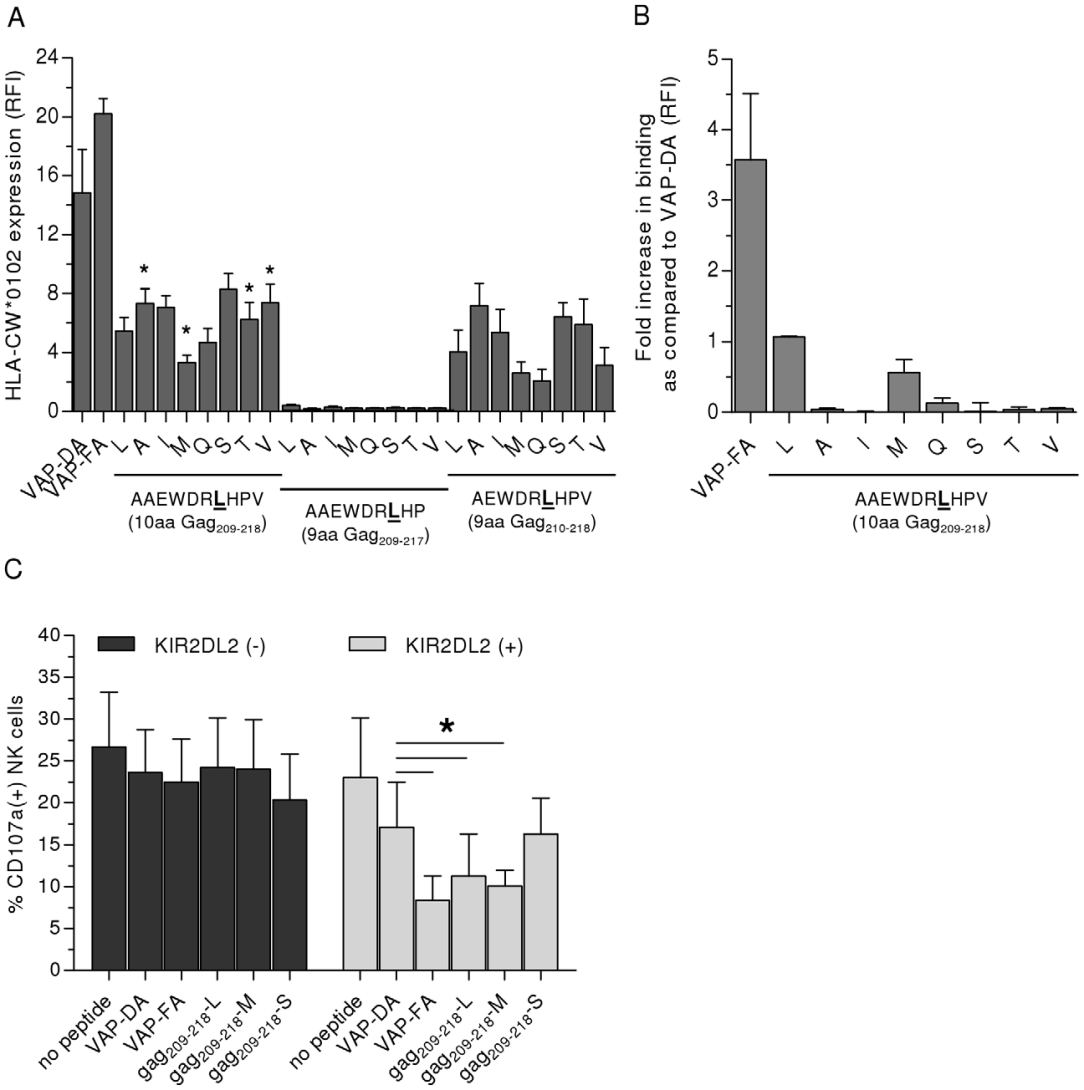
Ability of p24 Gag_209–218_ peptide variants for HLA-Cw*0102 stabilization and KIR2DL2-Fc binding. (**A**) Differential expression of HLA-Cw*0102 on T2 cells after co-incubation with peptide variants of HIV-1 p24 Gag_209–218_-L (AAEWDRLHPV). Peptide variants differed in length [9 or 10 amino acids (aa)] as well as in sequence (substitution of Leucine in position 7 with various amino acids). HLA-Cw*0102 expression is illustrated as relative median fluorescence intensity (RFI) as compared to unloaded T2 cells. Each column represents mean±SEM RFI of 4 independent experiments for each peptide variant. (**B**) Relative binding of KIR2DL2-Fc to T2 cells after co-incubation with selected HIV-1 p24 Gag_209–218_variants. KIR2DL2-Fc binding is illustrated as RFI as compared to VAP-DA-pulsed T2 cells. Each column represents mean±SEM RFI of 3 independent experiments for each 10 aa variant. (**C**) Different levels of NK cell degranulation after co-incubation with T2 cells in the presence of different p24 Gag_209–218_-L variants. Each column represents mean±SEM percentage of CD107a(+) NK cells of 4 different individuals. Unspecific degranulation of NK cells measured without target cells was deducted from each column. * indicates statistical significance as defined *p*<0.05.

The crystal structure and several functional studies suggested that requirements for sufficient engagement of KIR2DL2 do not only involve interactions between KIR2DL2 and HLA-C1 molecules but are also effected by amino acid residues in positions 7 and 8 (P7, P8) of the HLA-bound epitopes [15], [34]. We therefore assessed the sequence variations within Gag_209–218_ (AAEWDRLHPV) published in the Los Alamos HIV-1 sequence database, using more than 3,000 described HIV-1 sequences. While the amino acid residues in P1–6 and 9 (94.6–100%) as well as the Histidine (H) in P8 (>99.9%) of the epitope AAEWDRLHPV were highly conserved, sequence variations in P7 were observed in 29.2% of the published HIV-1 sequences. Variations included substitution of Leucine (L) to Alanine (A) (0.9%), Isoleucine (I) (3.6%), Methionine (M) (2.9%), Glutamine (Q) (0.1%), Serine (S) (0.1%), Threonine (T) (5.2%) and Valine (V) (16.3%). To determine the ability of these peptides containing sequence variations in P7 compared to the consensus sequence peptide to stabilize HLA-Cw*0102 and to enable binding to KIR2DL2, all peptide variants were synthesized (Table S4). In addition, as both 10aa and 9aa-long epitopes can be presented by HLA-Cw*0102, we also synthesized the 9aa long peptide variants lacking either the first or last amino acid (p24 Gag_210–218_-X, p24 Gag_209–217_-X). As depicted in Figure 4A, we observed p24 Gag_209–218_ variant peptides to display different abilities to stabilize HLA-Cw*0102 on T2 cells. The first and last amino acid of the 10 aa epitope were important for binding to HLA-Cw*0102, as no or reduced HLA stabilization was observed for these shorter 9-mer peptides. While lack of Alanine in P1 led to decreased expression of HLA-Cw*0102 as compared to the complementary 10 aa variants (−26.8±7.2% on average), absence of Valine in P10 caused almost complete loss of binding to HLA-Cw*0102 (−95±0.7% on average). Comparison between the individual 10 aa variants showed significant increased HLA-Cw*0102 expression in the presence of p24 Gag_209–218_-A, -V and –T as compared to the more frequently observed p24 Gag_209–218_-L variant (each *p*<0.05). In contrast, stabilization of HLA-Cw*0102 was decreased on p24 Gag_209–218_-M-loaded T2 cells (*p*<0.05).

In subsequent KIR binding assays we observed that all variants of p24 Gag_209–218_-L displayed decreased ability to bind KIR2DL2-Fc in complex with HLA-Cw*0102 as compared to the L variant (Figure 4B). Of note, p24 Gag_209–218_-M which caused the lowest HLA-Cw*0102 expression was the only variant that allowed for binding of KIR2DL2-Fc, however at lower levels than the L variant. In contrast, despite strong stabilization of HLA-Cw*0102 expression by the S, A and I variant, none of these allowed for binding of KIR2DL2 to HLA-Cw*0102. To confirm the functional relevance of these results from *in vitro* KIR binding assays, we performed NK cell degranulation assays using p24 Gag_209–218_-L, –M and -S variant peptides, and primary NK cells from *KIR2DL2(+)* individuals. The binding data for p24 Gag_209–218_-S were in line with the NK cell degranulation data, showing no significant inhibition of NK cell degranulation. Surprisingly, despite decreased ability to bind KIR2DL2-Fc*in vitro*, p24 Gag_209–218_-M-loaded T2 cells inhibited degranulation to the same degree as p24 Gag_209–218_-L (Figure 4C), suggesting that the weaker engagement of KIR2DL2 by p24 Gag_209–218_-M/HLA-Cw*0102 complexes might still be sufficient to prevent NK cell degranulation. Taken together, all 10-mer variants of p24 Gag_209–218_ containing a substitution of L in P7 stabilize HLA-Cw*0102 but with different abilities. However, aside from the consensus p24 Gag_209–218_-L peptide, p24 Gag_209–218_-M was the only variant peptide which allowed for KIR2DL2-Fc binding to HLA-Cw*0102 and affected degranulation of KIR2DL2-expressing primary NK cells.

## Discussion

To investigate the impact of sequence variations in HIV-1-derived peptides on KIR/HLA interactions and NK cell responses, we used a library of overlapping HIV-1 p24 peptides to identify peptides that stabilized HLA-Cw*0102 expression on TAP-deficient cells and assessed the consequences of these HIV-1 peptides for binding of the inhibitory NK cell receptor KIR2DL2 and for primary NK cell function. Although the majority of the OLPs were not able to stabilize HLA-Cw*0102, we identified 11 HIV-1 p24 Gag peptides which markedly stabilized HLA-Cw*0102 expression. In subsequent KIR binding and functional assays we observed that only the p24 Gag_209–218_ peptide was able to allow for KIR2DL2 binding to HLA-C and also significantly inhibited degranulation of KIR2DL2-expressing NK cells *in vitro*. Moreover, most naturally occurring variations at position 7 (P7) within the sequence of p24 Gag_209–218_ lead to abrogation of KIR2DL2 binding with the exception of p24 Gag_209–218_-M, which contained Methionine in P7. Taken together, we demonstrate that a HIV-1-derived peptide presented by HLA-Cw*0102 can be recognized by KIR2DL2(+) NK cells, providing new insights into the mechanisms that regulate NK cell function in HIV-1 infection.

HLA class I molecules expressed on infected cells present an array of self-peptides and virus-derived peptides, signaling to the host's immune system that the respective cell is infected. Viruses try to evade these host immune responses by a number of means, such as modulating HLA class I expression levels [35], encoding for inhibitory HLA-homologues [36], and selecting for sequence variations within HLA class I-presented viral peptides to escape T cell [37]–[41]or NK cell recognition [31], [42], [43]. Here, we used the interaction between KIR2DL2, an inhibitory NK cell receptor, and its ligand HLA-Cw*0102 as a model to study the consequences of sequence variations within HIV-1 p24 Gag peptides for KIR2DL2 binding and NK cell activity. Previous experimental and theoretical investigation of the peptides presented by HLA-Cw*0102 have led to the identification of several self and viral peptides, and the development of binding prediction models of various complexity [44]–[46]. In this study, we used HLA-Cw*0102-stabilization on TAP-deficient cell lines as a read-out for HLA binding, and identified 11 HIV-1 p24 peptides that stabilized HLA-Cw*0102. These peptides might serve as potential epitopes for CD8(+) T cell or NK cell recognition when presented by HLA-Cw*0102 molecules. Although all potential epitopes within these peptides were ranked in the highest quartile of the NetMHC and IEDB epitope prediction tools, only three OLPs (p24 Gag_168–177_:VI**P**MFSA**L**SE, p24 Gag_234–243_:SDI**A**GTTST**L**, p24 Gag_334–343_: LK**AL**GPAAT**L**)contained a conserved HLA-Cw*0102 binding motif as provided by the SYFPEITHI or Marsh2000 peptide binding prediction programs (x-[AL]-xxxxx-[L]; xx-[P]-xxxx-[L]) [47], [48]. Furthermore, predicted high binding affinities to HLA-Cw*0102 of other OLPs within the HIV-1 p24 protein could not be confirmed by our experimental approach. This suggests that binding affinities of peptides to HLA-Cw*0102 are complex and interaction of amino acids residues within the peptide might result in unique conformational structures allowing binding to HLA-Cw*0102 that cannot be accurately predicted by current binding prediction tools. Of the 11 identified HLA-Cw*0102-binding peptides, only peptide p24 Gag_168–177_ has been described previously to contain an HLA-Cw*0102-restricted epitope (VIPMFSAL) targeted by cytotoxic T-lymphocytes (CTLs) [49]. Notably, 7 of the 11 HIV-1 p24 peptides contained sequences for HLA-A or HLA-B-restricted epitopes listed in the CTL epitope summary of the Los Alamos Immunology database indicating potential cross-specificity between HLA-A/B and HLA-C molecules (p24 Gag_146–155, 162–171, 164–173, 209–218, 176–185,177–186, 334–343_). Presentation of viral epitopes by different classes of HLA molecules might serve as a mechanism to facilitate recognition of virus-infected cells by either NK cells, CTLs or both. Altogether, our results provided evidence for 10 new HLA-Cw*0102binding epitopes which would not have been predicted simply based on the currently defined HLA-Cw*0102 binding motif.

HLA-Cw*0102 represents an HLA-C group 1 molecule and can serve as a natural ligand for the inhibitory KIR2DL2 receptor [34], [50]. Assessment whether the newly identified HLA-Cw*0102-stabilizing p24 Gag epitopes allowed for binding of KIR2DL2 *in vitro* revealed that the majority of the identified peptides were not able to cause measurable KIR2DL2 binding to HLA-Cw*0102. In fact, only p24 Gag_209–218_ (AAEWDRLHPV) led to significant binding to KIR2DL2-Fc *in vitro*. These results were in line with the functional inhibition of KIR2DL2(+) NK cells measured by NK cell degranulation in the presence of p24 Gag_209–218_-pulsed T2 cells. Thus, we demonstrated that engagement of KIR2DL2 provided an inhibitory signal which was sufficient to prevent degranulation in primary KIR2DL2(+) NK cells. In contrast, results obtained with HLA-Cw*0102-presented peptides which did not allow for KIR2DL2 binding suggest that lack of a KIR2DL2-mediated inhibitory signal might increase vulnerability of cells for NK cell-mediated killing. In this context, previous investigations of the peptide-dependent interaction of KIR2DL2 with HLA-C group 1 molecules are noteworthy. Several studies emphasized the importance of peptide residues P7 and P8 regarding binding affinities to KIR2DL2 [15], [23]–[28]. Comparison of the amino acid residues in P7 and P8 of the 11 peptides that stabilized HLA-Cw*0102 revealed marked similarities regarding the amino acid residues in position 7 but not in P8. Similar to p24 Gag_209–218_ (AAEWDRLHPV) five of the naturally occurring HIV peptides contained amino acids at P7 with non-polar hydrophobic residues. No polar-charged or basic amino acid residues were found in P7 of the peptides implementing a motif which could be rather favorable for stabilization of HLA-Cw*0102. In contrast, p24 Gag_209–218_ was the only peptide containing a basic amino acid in P8 (Histidine) suggesting either a potential primary interaction of the Histidine with the KIR2DL2 molecule or a different overall structure of the peptide/HLA-Cw*0102 complex that promoted binding to KIR2DL2. Surprisingly, based on the crystal structure of KIR2DL2 in complex with peptide-bound HLA-Cw3, Boyington *et al*. predicted KIR2DL2 binding only to be allowed in the presence of amino acid residues that are not larger than Serine or Threonine in P8 [15]. However, the amino acid side chain of Histidine is larger, indicating a potential differential conformation in complex with HLA-Cw*0102 as compared to HLA-Cw3.

We also investigated the impact of alterations in P7 within the p24 Gag_209–218_ peptide on HLA-Cw*0102-stabilization as well as on the ability to bind to KIR2DL2. Our results identified the first and last amino acids of AAEWDRLHPV to be important for HLA-Cw*0102 stabilization. In particular, lack of the last uncharged amino acid resulted in complete loss of HLA-Cw*0102 stabilization. The hydrophobic side chains in P1/(P2) and P10 therefore might support anchoring the peptide in the HLA-Cw*0102 binding grove. Of note, substitution of the Leucine in P7 also affected stabilization of HLA-Cw*0102, indicating a yet underestimated role of P7 in defining HLA binding specificity. However, independent of the impact on HLA-Cw*0102 stabilization, alterations of the peptide sequence in P7 led to complete abrogation of KIR2DL2 binding to peptide-bound HLA-Cw*0102 for most described naturally occurring sequence changes in that position. This was also the case for substitutions with similar hydrophobic amino acids like Alanine, Isoleucine or Valine, but that this was not the case for Methionine suggests a potential similar conformation as compared to Leucine. Taken together, our observations confirm the crucial role of peptide position 7 in the interaction of KIR2DL2 and HLA-Cw*0102 molecules, but were not entirely in line with the predictions derived from the crystal structure of KIR2DL2 in complex with HLA-Cw3 [15]. Thus, KIR2DL2/HLA-Cw*0102 interactions might demand different requirements to the sequence of the bound peptide as compared to other HLA-C group 1 molecules, potentially resulting in increased specificity.

The described peptide-specificity of the interaction between HLA-C group 1 molecules and KIR2DL2 might affect the ability of NK cells to recognize and lyse virus-infected cells, modulating the degree of antiviral immune pressure, and potentially resulting in viral evolution to evade NK cell mediated immunity. We recently described that viral sequence polymorphisms associated with the expression of specific KIR alleles can be detected in the HIV-1 sequence on a population level, providing the first evidence for NK-cell mediated immune pressure and viral escape in HIV-1 infection [14]. These data suggested that viral escape mutations within the HIV-1 proteome can lead to the engagement of inhibitory KIRs, providing an inhibitory signal to NK cells and preventing NK cell-mediated killing of infected cells. A potential shift toward HLA class I-presented epitopes that do not allow for engagement of inhibitory KIRs might shift the balance toward NK cell activation, and elimination of infected cells. However, due to the small sample size within that initial study, the HLA class I molecules that might have presented the HIV-1 epitope variants to inhibitory KIRs were not identified. In the current study we demonstrate that the majority of the HLA-Cw*0102-presented HIV-1 clade B consensus epitopes within p24 Gag do not allow for the binding of KIR2DL2, and do not result in the inhibition of KIR2DL2(+) NK cells. However, for the one HLA-Cw*0102-restricted epitope in p24 Gag that allowed for KIR2DL2 binding (p24 Gag_209–218_), the majority of published HIV-1 sequences encoded the Leucine in position 7 that showed the strongest ability to bind KIR2DL2 in complex with HLA-Cw*0102, despite several naturally occurring sequence variations in that position. Notably, we observed an enrichment of Leucine in position 7 (p24 Gag_215_) in HIV-1 sequences of individuals with the compounded HLA-Cw*0102/KIR2DL2 using the previously published HIV-1 clade B sequences of 91 chronically HIV-1infected individuals [14]. While all HIV-1 sequences of individuals encoding for HLA-Cw*0102/KIR2DL2 (5/5) contained Leucine at Gag_215_, the proportion of Leucine at that position was decreased to 75% (65/86) in individuals who did not encode for this genotype. However, due to the limited number of HLA-Cw*0102/KIR2DL2-positive individuals in this cohort this effect did not reach statistical significance (*p* = 0.33; Fisher's exact probability test, two-tailed). Whether the relative conservation of the Leucine in P7 of the p24 Gag_209–218_ sequence over other amino acids that did not result in binding of KIR2DL2 represents the consequence of HIV-1 escape from KIR2DL2(+) NK cell responses requires further elucidation.

Taken together, we performed a comprehensive screening for the peptides within HIV-1 p24 Gag that stabilized HLA-Cw*0102 and allowed for the binding of the inhibitory NK cell receptor KIR2DL2, and identified peptide p24 Gag_209–218_ which allowed for both binding of KIR2DL2 and inhibition of primary KIR2DL2(+) NK cell function *in vitro*. Although these initial investigations of KIR2DL2/HLA interactions were limited to only one HLA/KIR interaction, the systematic approach described here provided new insights in the complex interaction between KIR molecules and HLA/peptide complexes. Furthermore, the results of this study can act as a foundation to further elucidate the role of variations within HIV-1 epitopes on HLA/KIR interactions, and the ability of viruses to evade NK cell-mediated immune pressure. Selecting for sequence polymorphisms within HLA class I presented epitopes that allow for the engagement of inhibitory KIRs on NK cells might represent a means for HIV-1 to evade NK cell recognition.

## Materials and Methods

### Human blood samples and cell lines

The TAP-deficient cell line T2 (homozygous for HLA-A*0201, B*51, Cw*0102) was used for all the experiments assessing peptide binding to HLA class I, binding of KIR, and functional responses by primary NK cells [32], [33], [51]. T2 cells were cultured in R10 medium [RPMI medium 1640 (Sigma) supplemented with 1% (v/v) penicillin/streptomycin (Cellgro) and 10% (v/v) Fetal Calf Serum (FCS) (Sigma)].

### Ethics statement

Furthermore, a total of 6healthy donors were enrolled in this study for the functional studies of primary NK cells. All subjects were recruited at Massachusetts General Hospital (MGH), and gave written informed consent for participation in this study. The study was approved by the Partners Institutional Review Board (IRB)(2010P002121).

### Antibodies

The following antibodies were used for the flow cytometric analysis of T2 cells: anti-human HLA-A/B/C-PE (clone W6/32; eBioscience), mouse IgG2a-PE Isotype control (eBioscience), mouse anti-human HLA-C (DT9, kindly provided by Dr. Mary Carrington) and anti-mouse IgG-PE (Sigma).Flow cytometric identification and analysis of NK cells was performed by using anti-human CD56-Alexa Fluor 700, anti-human CD3-Pacific Blue, anti-human CD16-APC-Cy7, anti-human CD107a-PE-Cy5 (all BD Pharmingen) and anti-human KIR2DL2/L3-PE (Miltenyi Biotec).

### Peptide-induced HLA stabilization and analysis of HLA expression

A total of 217 decameric peptides spanning the HIV-1 p24 Gag (consensus B) sequence and overlapping by 9 amino acids (aa) were studied for HLA-Cw*0102 stabilization. Peptide-induced HLA stabilization was assessed by culturing T2 cells in the presence or absence of individual overlapping HIV-1 p24 peptides (OLPs) or two control peptides (VAP-DA: VAPWNSDAL, VAP-FA: VAPWNSFAL) that had been previously shown to bind to HLA-Cw*0102 [23]. 2*10^5^ T2 cells were cultured overnight in RPMI medium 1640 in 96 well round-bottom plates at 26°C. Individual peptides were added at a final concentration of 0.04 mg/ml. After co-incubation, T2 cells were washed and then either stained for HLA-A/B/C (clone W6/32) or HLA-C (DT9), respectively. After fixation in 4% (w/v) paraformaldehyde (Affymetrix) cells were analyzed by flow cytometry.

### KIR2DL2 binding assay

KIR2D2L2-IgGand KIR3DS1-IgG fusion constructs (KIR2DL2-Fc & KIR3DS1-Fc Chimera; R&D Systems) were conjugated with protein A Alexa Fluor 488 (Invitrogen) in a molar ratio of 6∶1. T2 cells (2*10^5^) were incubated with 100 µM peptide as described above and then stained with KIR-Fc. After fixation in 4% (w/v) paraformaldehyde cells were analyzed by flow cytometry.

### NK cell degranulation assay

Assessment of primary NK cell degranulation was performed using peripheral blood mononuclear cells (PBMC) from *KIR2DL2*(+) healthy donors. PBMC were isolated from peripheral blood samples by density gradient centrifugation using Histopaque-1077 (Sigma) and then incubated overnight in R10 medium supplemented with 1 ng/ml IL-15 (Cellgro). PBMC (5*10^5^ cells) were co-incubated with peptide-pulsed T2 cells (1*10^5^) cells at an effector-to-target (E∶T) ratio of 5∶1 in R10 medium in 96 well V-bottom plates (Corning) containing anti-human CD107a (20 µl/ml). After 1 hour incubation at 26°C, monensin (6 µl/mL, BD GolgiStop) was added followed by additional 3 hours incubation. Cells were then stained with anti-CD3, anti-CD16, anti-CD56 and anti-KIR2DL2/3. After fixation in 4% (w/v) paraformaldehyde cells were analyzed by flow cytometry.

### Data acquisition, analysis and statistical analysis

Acquisition of flow cytometric data was performed on a BD LSRFortessa (BD Biosciences) and then analyzed using FlowJo software v7.6.5 (Tree Star, Inc). Scale of the x-axis of overlaid histograms is displayed as percentage of the maximum (% of Max). Calculation of % of Max was done by dividing the number of cells in each bin by the number of cells in the bin that contains the largest number of cells. Calculation of relative fluorescence intensity (RFI) was based on median fluorescence intensity (MFI) and was performed as follows: [MFI (peptide-loaded T2 cells)/MFI (unloaded T2 cells)] -1. Values in bar graphs are presented as mean±SEM unless stated otherwise. Statistical analysis was performed using GraphPad Prism 5.0 (GraphPad Software, Inc.).Statistical comparison between groups was performed using repeated measures ANOVA. Epitope prediction within the HIV-1 p24 protein was performed using NetMHC (http://www.cbs.dtu.dk/services/NetMHC-3.0/) and IEDB MHC-I binding predictions (http://tools.immuneepitope.org/analyze/html/mhc_binding.html).

## Supporting Information

Figure S1 **Gating strategies.** (**A**) Representative dot plots of T2 cell gating strategy. T2 cells were defined by Forward (FSC-Area) and Sideward Scatter (SSC-Area). In a subsequent gate single cells were distinguished from doublets using Forward-Scatter (FSC-Width). (**B**) Representative dot plots of NK cell gating strategy. Lymphocytes were defined by Forward (FSC-Area) and Sideward Scatter (SSC-Area). In a subsequent gate CD3(−) single cells were distinguished from doublets and T cells using Forward-Scatter (FSC-Width) and CD3. NK cells were then defined as either CD16(+) or CD56(+) and further discriminated into KIR2DL2/3(−) and KIR2DL2/3(+) cells. Degranulation of NK cells was measured by expression of CD107a.
(TIF)

Figure S2 **Saturation curves.** The figure shows specific stabilization of HLA-A/B/C (**A**) and HLA-Cw*0102 (**B**) on the surface of T2 cells after co-incubation with increasing concentrations of HIV-1 p24 Gag_209–218_. T2 cells were pulsed overnight with HIV-1 p24 Gag_209–218_at concentrations between 1 and 200 µg/ml and then stained with an HLA-A/B/C-specific antibody (clone W6/32) and an HLA-C-specific antibody (DT9) respectively. HLA expression is illustrated as relative median fluorescence intensity (RFI) as compared to unloaded T2 cells. (**C**) Figure 1C illustrates specific binding of KIR2DL2-Fc to peptide-loaded T2 cells. T2 cells were loaded overnight with HIV-1 p24 Gag_209–218_or VAP-FA at a concentration of 100 µM and then stained with increasing concentrations of KIR2DL2-Fc. Binding is displayed relative to binding of VAP-DA loaded T2 cells.
(TIF)

Table S1 **HLA expression of HIV-1 p24 peptide-pulsed T2 cells.** The table shows the mean HLA expression of T2 cells after co-incubation with the respective HIV-1 p24 overlapping peptide. HLA expression was assessed using the HLA-A/B/C-specific antibody W6/32 and is illustrated as mean of the relative median fluorescence intensity (RFI) of three independent experiments. Peptides leading to marked stabilization of HLA class I expression by more than 30% compared to un-pulsed T2 cells are shown in gray and where selected for further analysis of HLA-Cw*0102-specific stabilization.
(PDF)

Table S2 **Epitope prediction within HIV-1 p24 consensus sequence (clade B).** The table shows results of the epitope prediction for HLA-Cw*0102 using the NetMHC3.0 and the IEDB MHC-I binding prediction tool. Displayed are the percentile ranks of epitopes within the 11 selected HIV-1 p24 overlapping peptides which showed the highest stabilization of HLA-Cw*0102 in the *in vitro* HLA stabilization experiment. Percentile rank determined by the 217 overlapping peptides included in this analysis.
(PDF)

Table S3 ***HLA*** **and** ***KIR*** **genotypes of individuals.** The table shows the *HLA class I* and *KIR* genotypes of individuals enrolled in the present study. ^a),b)^ indicate whether NK cells derived from these individuals were used in a degranulation assay displayed in either Figure 3B or Figure 4B. 1 indicates the presence of the respective gene, 0 the absence respectively.
(PDF)

Table S4 **HIV-1 p24 Gag_209–218_-L peptide variants.** The table displays the sequence of synthesized peptide variants based on the sequence variations in position Gag_215_ published in the Los Alamos HIV-1 sequence database describing more than 3,000 HIV-1 sequences.
(PDF)
