# Supplementary figures and images for: HLA-Cw*0102-Restricted HIV-1 p24 Epitope Variants Can Modulate the Binding of the Inhibitory KIR2DL2 Receptor and Primary NK Cell Function
Source: PLoS Pathog. 2012 Jul 12;8(7):e1002805. doi: 10.1371/journal.ppat.1002805 (PMC3395618; doi:10.1371/journal.ppat.1002805)

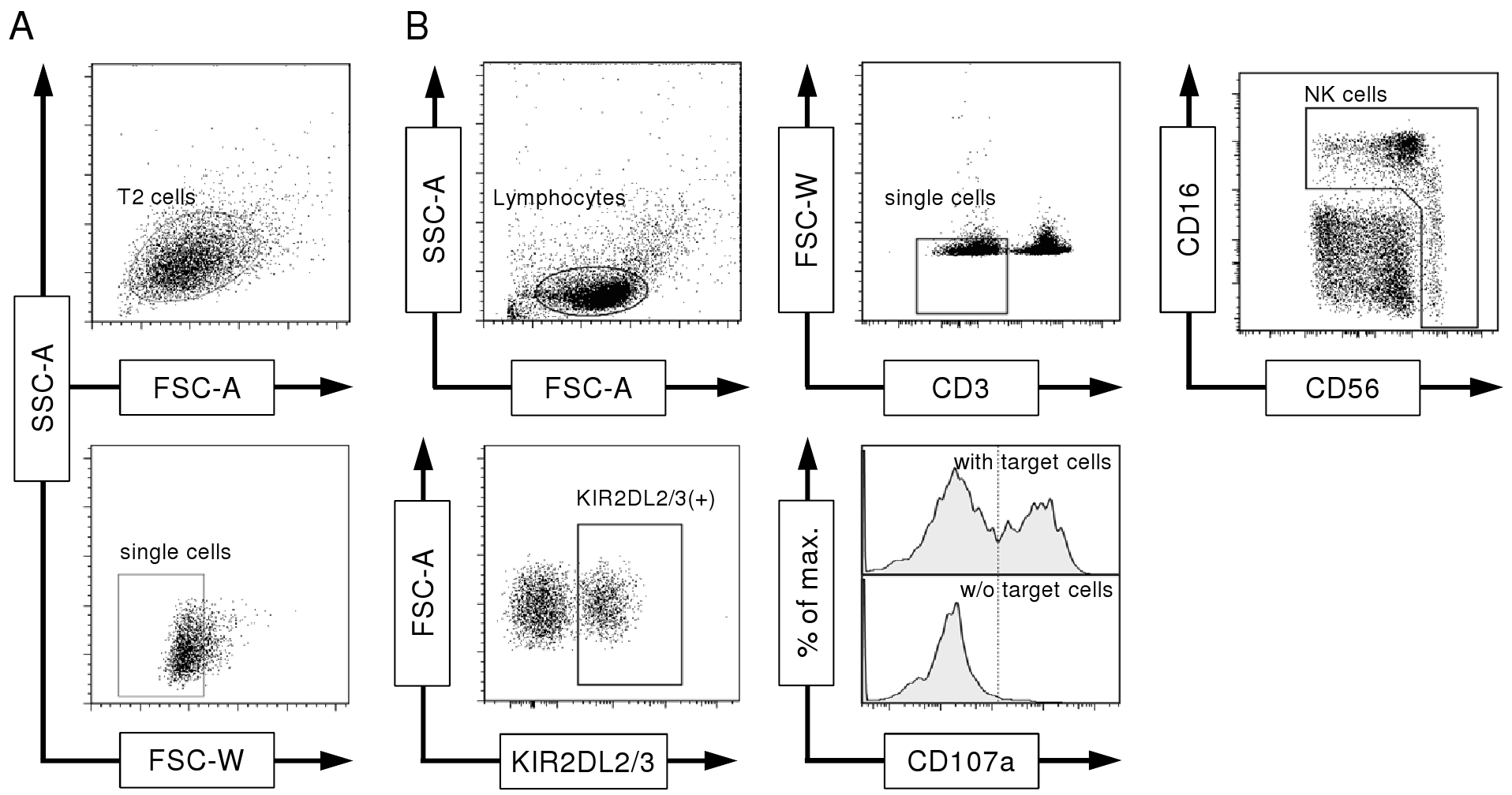

Supplement: Figure S1 — Gating strategies. (A) Representative dot plots of T2 cell gating strategy. T2 cells were defined by Forward (FSC-Area) and Sideward Scatter (SSC-Area). In a subsequent gate single cells were distinguished from doublets using Forward-Scatter (FSC-Width). (B) Representative dot plots of NK cell gating strategy. Lymphocytes were defined by Forward (FSC-Area) and Sideward Scatter (SSC-Area). In a subsequent gate CD3(−) single cells were distinguished from doublets and T cells using Forward-Scatter (FSC-Width) and CD3. NK cells were then defined as either CD16(+) or CD56(+) and further discriminated into KIR2DL2/3(−) and KIR2DL2/3(+) cells. Degranulation of NK cells was measured by expression of CD107a. (TIF) [file ppat.1002805.s001.tif]

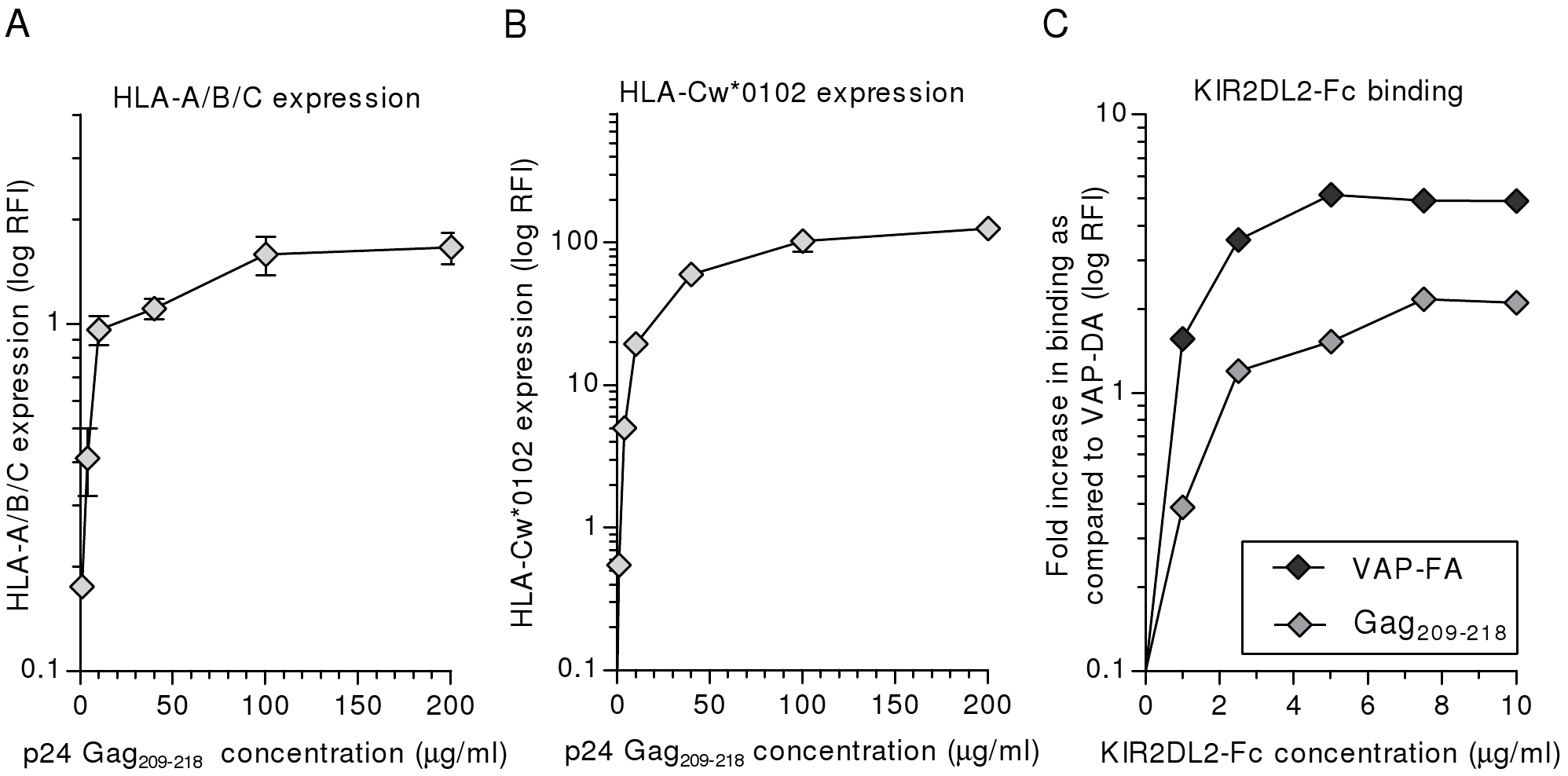

Supplement: Figure S2 — Saturation curves. The figure shows specific stabilization of HLA-A/B/C (A) and HLA-Cw*0102 (B) on the surface of T2 cells after co-incubation with increasing concentrations of HIV-1 p24 Gag209–218. T2 cells were pulsed overnight with HIV-1 p24 Gag209–218at concentrations between 1 and 200 µg/ml and then stained with an HLA-A/B/C-specific antibody (clone W6/32) and an HLA-C-specific antibody (DT9) respectively. HLA expression is illustrated as relative median fluorescence intensity (RFI) as compared to unloaded T2 cells. (C) Figure 1C illustrates specific binding of KIR2DL2-Fc to peptide-loaded T2 cells. T2 cells were loaded overnight with HIV-1 p24 Gag209–218or VAP-FA at a concentration of 100 µM and then stained with increasing concentrations of KIR2DL2-Fc. Binding is displayed relative to binding of VAP-DA loaded T2 cells. (TIF) [file ppat.1002805.s002.tif]
