# Supplementary material for: HLA-Cw*0102-Restricted HIV-1 p24 Epitope Variants Can Modulate the Binding of the Inhibitory KIR2DL2 Receptor and Primary NK Cell Function
Source: PLoS Pathog. 2012 Jul 12;8(7):e1002805. doi: 10.1371/journal.ppat.1002805 (PMC3395618; doi:10.1371/journal.ppat.1002805)
Supplement: Table S1 — HLA expression of HIV-1 p24 peptide-pulsed T2 cells. The table shows the mean HLA expression of T2 cells after co-incubation with the respective HIV-1 p24 overlapping peptide. HLA expression was assessed using the HLA-A/B/C-specific antibody W6/32 and is illustrated as mean of the relative median fluorescence intensity (RFI) of three independent experiments. Peptides leading to marked stabilization of HLA class I expression by more than 30% compared to un-pulsed T2 cells are shown in gray and where selected for further analysis of HLA-Cw*0102-specific stabilization. (PDF) [file ppat.1002805.s003.pdf]

Table S1: HLA expression of HIV-1 p24 peptide-pulsed T2 cells.

No.[a)]	Sequence [b)]	RFI [c)]	No.[a)]	Sequence [b)]	RFI [c)]	No.[a)]	Sequence [b)]	RFI [c)]	No.[a)]	Sequence [b)]	RFI [c)]
1	PIVQNLQGQM	<0.01	56	LNTVGGHQAA	0.26	111	LQEQIGWMTN	0.12	166	DRFYKTLRAE	<0.01
2	IVQNLQGQMV	0.57	57	NTVGGHQAAM	0.10	112	QEQIGWMTNN	0.06	167	RFYKTLRAEQ	<0.01
3	VQNLQGQMVH	0.31	58	TVGGHQAAMQ	0.14	113	EQIGWMTNNP	0.01	168	FYKTLRAEQA	<0.01
4	QNLQGQMVHQ	0.02	59	VGGHQAAMQM	0.07	114	QIGWMTNNPP	0.37	169	YKTLRAEQAS	0.18
5	NLQGQMVHQA	0.22	60	GGHQAAMQML	0.10	115	IGWMTNNPPI	0.26	170	KTLRAEQASQ	0.01
6	LQGQMVHQAI	0.48	61	GHQAAMQMLK	0.02	116	GWMTNNPPIP	0.95	171	TLRAEQASQE	<0.01
7	QGQMVHQAIS	<0.01	62	HQAAMQMLKE	0.13	117	WMTNNPPIPV	0.16	172	LRAEQASQEV	<0.01
8	GQMVHQAISP	0.26	63	QAAMQMLKET	0.12	118	MTNNPPIPVG	0.45	173	RAEQASQEVK	0.03
9	QMVHQAISPR	0.19	64	AAMQMLKETI	0.34	119	TNNPPIPVGE	<0.01	174	AEQASQEVKN	<0.01
10	MVHQAISPRT	0.16	65	AMQMLKETIN	0.04	120	NNPPIPVGEI	0.39	175	EQASQEVKNW	<0.01
11	VHQAISPRTL	0.59	66	MQMLKETINE	<0.01	121	NPPIPVGEIY	0.13	176	QASQEVKNWM	<0.01
12	HQAISPRTLN	<0.01	67	QMLKETINEE	0.07	122	PPIPVGEIYK	0.11	177	ASQEVKNWMT	0.03
13	QAISPRTLNA	0.00	68	MLKETINEEA	0.25	123	PIPVGEIYKR	0.01	178	SQEVKNWMTE	<0.01
14	AISPRTLNAW	0.87	69	LKETINEEAA	0.41	124	IPVGEIYKRW	<0.01	179	QEVKNWMTET	0.02
15	ISPRTLNAWV	0.62	70	KETINEEAAE	0.24	125	PVGEIYKRWI	0.19	180	EVKNWMTETL	0.08
16	SPRTLNAWVK	0.21	71	ETINEEAAEW	0.12	126	VGEIYKRWII	<0.01	181	VKNWMTETLL	<0.01
17	PRTLNAWVKV	0.10	72	TINEEAAEWD	0.23	127	GEIYKRWIIL	0.04	182	KNWMTETLLV	0.16
18	RTLNAWVKVV	0.75	73	INEEAAEWDR	0.06	128	EIYKRWIILG	0.06	183	NWMTETLLVQ	<0.01
19	TLNAWVKVVE	0.23	74	NEEAAEWDRL	0.13	129	IYKRWIILGL	0.02	184	WMTETLLVQN	<0.01
20	LNAWVKVVEE	0.14	75	EEAAEWDRLH	0.17	130	YKRWIILGLN	0.88	185	MTETLLVQNA	0.21
21	NAWVKVVEEK	0.32	76	EAAEWDRLHP	1.08	131	KRWIILGLNK	0.08	186	TETLLVQNAN	0.43
22	AWVKVVEEKA	0.09	77	AAEWDRLHPV	0.81	132	RWIILGLNKI	0.51	187	ETLLVQNANP	0.18
23	WVKVVEEKAF	0.14	78	AEWDRLHPVH	1.19	133	WIILGLNKIV	0.70	188	TLLVQNANPD	0.26
24	VKVVEEKAFS	0.13	79	EWDRLHPVHA	0.32	134	IILGLNKIVR	0.15	189	LLVQNANPDC	0.08
25	KVVEEKAFSP	0.16	80	WDRLHPVHAG	0.21	135	ILGLNKIVRM	0.32	190	LVQNANPDCK	0.08
26	VVEEKAFSPE	0.25	81	DRLHPVHAGP	0.37	136	LGLNKIVRMY	0.29	191	VQNANPDCKT	0.22
27	VEEKAFSPEV	0.23	82	RLHPVHAGPI	0.67	137	GLNKIVRMYS	0.34	192	QNANPDCKTI	0.58
28	EEKAFSPEVI	0.16	83	LHPVHAGPIA	0.37	138	LNKIVRMYSP	0.00	193	NANPDCKTIL	<0.01
29	EKAFSPEVIP	0.30	84	HPVHAGPIAP	0.11	139	NKIVRMYSPT	0.06	194	ANPDCKTILK	<0.01
30	KAFSPEVIPM	0.82	85	PVHAGPIAPG	0.14	140	KIVRMYSPTS	<0.01	195	NPDCKTILKA	<0.01
31	AFSPEVIPMF	0.42	86	VHAGPIAPGQ	0.13	141	IVRMYSPTSI	<0.01	196	PDCKTILKAL	0.13
32	FSPEVIPMFS	0.52	87	HAGPIAPGQM	0.31	142	VRMYSPTSIL	0.68	197	DCKTILKALG	0.07
33	SPEVIPMFSA	0.47	88	AGPIAPGQMR	0.00	143	RMYSPTSILD	0.11	198	CKTILKALGP	<0.01
34	PEVIPMFSAL	0.73	89	GPIAPGQMRE	0.01	144	MYSPTSILDI	1.15	199	KTILKALGPA	0.35
35	EVIPMFSALS	0.87	90	PIAPGQMREP	0.03	145	YSPTSILDIR	0.54	200	TILKALGPAA	0.57
36	VIPMFSALSE	1.02	91	IAPGQMREPR	<0.01	146	SPTSILDIRQ	0.06	201	ILKALGPAAT	0.18
37	IPMFSALSEG	0.34	92	APGQMREPRG	<0.01	147	PTSILDIRQG	0.06	202	LKALGPAATL	0.81
38	PMFSALSEGA	0.51	93	PGQMREPRGS	<0.01	148	TSILDIRQGP	<0.01	203	KALGPAATLE	0.12
39	MFSALSEGAT	0.14	94	GQMREPRGSD	<0.01	149	SILDIRQGPK	0.16	204	ALGPAATLEE	0.07
40	FSALSEGATP	<0.01	95	QMREPRGSDI	<0.01	150	ILDIRQGPKE	<0.01	205	LGPAATLEEM	<0.01
41	SALSEGATPQ	0.19	96	MREPRGSDIA	<0.01	151	LDIRQGPKEP	<0.01	206	GPAATLEEMM	<0.01
42	ALSEGATPQD	0.21	97	REPRGSDIAG	0.12	152	DIRQGPKEPF	<0.01	207	PAATLEEMMT	<0.01
43	LSEGATPQDL	<0.01	98	EPRGSDIAGT	0.25	153	IRQGPKEPFR	<0.01	208	AATLEEMMTA	<0.01
44	SEGATPQDLN	<0.01	99	PRGSDIAGTT	<0.01	154	RQGPKEPFRD	<0.01	209	ATLEEMMTAC	<0.01
45	EGATPQDLNT	<0.01	100	RGSDIAGTTS	0.18	155	QGPKEPFRDY	<0.01	210	TLEEMMTACQ	<0.01
46	GATPQDLNTM	<0.01	101	GSDIAGTTST	0.15	156	GPKEPFRDYV	<0.01	211	LEEMMTACQG	<0.01
47	ATPQDLNTML	0.16	102	SDIAGTTSTL	1.18	157	PKEPFRDYVD	<0.01	212	EEMMTACQGV	0.44
48	TPQDLNTMLN	0.05	103	DIAGTTSTLQ	0.84	158	KEPFRDYVDR	<0.01	213	EMMTACQGVG	0.65
49	PQDLNTMLNT	0.05	104	IAGTTSTLQE	0.82	159	EPFRDYVDRF	<0.01	214	MMTACQGVGG	0.57
50	QDLNTMLNTV	0.28	105	AGTTSTLQEQ	0.63	160	PFRDYVDRFY	<0.01	215	MTACQGVGGP	0.37
51	DLNTMLNTVG	0.10	106	GTTSTLQEQI	0.75	161	FRDYVDRFYK	<0.01	216	TACQGVGGPG	0.21
52	LNTMLNTVGG	<0.01	107	TTSTLQEQIG	0.35	162	RDYVDRFYKT	<0.01	217	ACQGVGGPGH	<0.01
53	NTMLNTVGGH	0.67	108	TSTLQEQIGW	0.58	163	DYVDRFYKTL	<0.01		VAP-DA	1.54
54	TMLNTVGGHQ	0.24	109	STLQEQIGWM	0.51	164	YVDRFYKTLR	<0.01		VAP-FA	1.23
55	MLNTVGGHQA	0.27	110	TLQEQIGWMT	0.46	165	VDRFYKTLRA	<0.01			

a) Number of the HIV-1 p24 overlapping peptide (p24 OLP #), b) amino acid sequence of the peptide,
c) RFI (relative fluorescence intensity) as compared to unloaded T2 cells presented as mean from 3 independent experiments.
