# Supplementary material for: HLA-Cw*0102-Restricted HIV-1 p24 Epitope Variants Can Modulate the Binding of the Inhibitory KIR2DL2 Receptor and Primary NK Cell Function
Source: PLoS Pathog. 2012 Jul 12;8(7):e1002805. doi: 10.1371/journal.ppat.1002805 (PMC3395618; doi:10.1371/journal.ppat.1002805)
Supplement: Table S2 — Epitope prediction within HIV-1 p24 consensus sequence (clade B). The table shows results of the epitope prediction for HLA-Cw*0102 using the NetMHC3.0 and the IEDB MHC-I binding prediction tool. Displayed are the percentile ranks of epitopes within the 11 selected HIV-1 p24 overlapping peptides which showed the highest stabilization of HLA-Cw*0102 in the in vitro HLA stabilization experiment. Percentile rank determined by the 217 overlapping peptides included in this analysis. (PDF) [file ppat.1002805.s004.pdf]

Table S2: Epitope prediction within HIV-1 p24 consensus sequence (clade B).

Prediction tool: NetMHC 3.0					
p24 OLP	Sequence	Length	Percentile rank	Affinity	Prediction*
30, 32	FSPEVIPM	8	0.5	14.83	Strong
36	VIPMFSAL	8	0.9	11.55	Weak binder
144, 145	YSPTSILDI	9	1.4	11.18	Weak binder
77	AAEWDRLHPV	10	1.8	10.75	Weak binder
102	IAGTTSTL	8	2.7	9.71	-
14	ISPRTLNA	8	5	6.73	-
82	LHPVHAGPI	9	7.8	3.92	-
202	ALGPAATL	8	13.2	2.17	-
106	GTTSTLQEQI	10	26	-1.43	-
# identified HLA-Cw*0102-stabilizing OLP containing the predicted epitope, * Strong binder threshold 14.32. Weak binder threshold score 9.06.					

Prediction tool: IEDB MHC-I binding prediction				
p24 OLP	Sequence	Length	Percentile rank	
36	VIPMFSAL	8	0.6	
82	RLHPVHAGPI	10	0.7	
102	IAGTTSTL	8	1.4	
30	KAFSPEVIPM	10	1.4	
32	FSPEVIPM	8	1.6	
77	AEWDRLHPV	9	2.7	
144	MYSPTSIL	8	5.7	
145	YSPTSILDI	9	6.6	
202	LKALGPAATL	10	9.6	
14	SPRTLNAW	8	8.5	
106	GTTSTLQEQI	10	27	
# identified HLA-Cw*0102-stabilizing OLP containing the predicted epitope				
