# Supplementary material for: HLA-Cw*0102-Restricted HIV-1 p24 Epitope Variants Can Modulate the Binding of the Inhibitory KIR2DL2 Receptor and Primary NK Cell Function
Source: PLoS Pathog. 2012 Jul 12;8(7):e1002805. doi: 10.1371/journal.ppat.1002805 (PMC3395618; doi:10.1371/journal.ppat.1002805)
Supplement: Table S3 — HLA and KIR genotypes of individuals. The table shows the HLA class I and KIR genotypes of individuals enrolled in the present study. a),b) indicate whether NK cells derived from these individuals were used in a degranulation assay displayed in either Figure 3B or Figure 4B. 1 indicates the presence of the respective gene, 0 the absence respectively. (PDF) [file ppat.1002805.s005.pdf]

Table S3: HLA and KIR genotypes of individuals

Donor#	1	2	3 [a,b]	4 [a,b]	5 [a,b]	6 [b]
HLA-A	0101/3101	0201/6801	0201/0301	0201/0301	2601/3101	3402/7400
HLA-B	4403/5701	4402/5101	0702/0702	0702/5801	3501/3801	0705/4403
HLA-C	0401/0602	0501/1402	0702/0702	0701/0702	0401/1203	0401/0702
KIR2DL1	1	1	1	1	1	1
KIR2DL2	1	1	1	1	1	1
KIR2DL3	1	1	1	1	1	1
KIR2DL4	1	1	1	1	1	1
KIR2DL5	1	1	1	0	1	1
KIR2DS1	0	1	1	0	0	1
KIR2DS2	1	1	1	1	1	1
KIR2DS3	1	0	0	0	1	1
KIR2DS4 -A1	197	219	197	197	197	219
KIR2DS4 - A2	0	0	0	0	0	0
KIR2DS5	0	1	1	0	0	0
KIR2DP1	1	1	1	1	1	1
KIR3DL1	1	1	1	1	1	1
KIR3DS1	0	1	1	0	0	1
KIR3DL2	1	1	1	1	1	1
KIR3DL3	1	1	1	1	1	1
KIR2DL2/3 (+) [#]	21.00%	33.20%	38.00%	35.30%	16.50%	18.50%

[a)] data displayed in figure 3B; [b)] data displayed in figure 4B; [#] percentage of KIR2DL2/3(+) NK cells. KIR genotype: 1 indicates the presence of the respective gene, 0 the absence respectively.
