# Supplementary material for: HLA-Cw*0102-Restricted HIV-1 p24 Epitope Variants Can Modulate the Binding of the Inhibitory KIR2DL2 Receptor and Primary NK Cell Function
Source: PLoS Pathog. 2012 Jul 12;8(7):e1002805. doi: 10.1371/journal.ppat.1002805 (PMC3395618; doi:10.1371/journal.ppat.1002805)
Supplement: Table S4 — HIV-1 p24 Gag209–218-L peptide variants. The table displays the sequence of synthesized peptide variants based on the sequence variations in position Gag215 published in the Los Alamos HIV-1 sequence database describing more than 3,000 HIV-1 sequences. (PDF) [file ppat.1002805.s006.pdf]

Table S4: p24 $Gag_{209-218}$-L peptide variants

Name	Sequence	aa substitution	Length
$Gag_{209-218}$-L	AAEWDRLHPV	-	10
$Gag_{209-218}$-A	AAEWDRAHPV	A	10
$Gag_{209-218}$-I	AAEWDRIHPV	I	10
$Gag_{209-218}$-M	AAEWDRMHPV	M	10
$Gag_{209-218}$-Q	AAEWDRQHPV	Q	10
$Gag_{209-218}$-S	AAEWDRSHPV	S	10
$Gag_{209-218}$-T	AAEWDRTHPV	T	10
$Gag_{209-218}$-V	AAEWDRVHPV	V	10
$Gag_{209-217}$-L	AAEWDRLHP	-	9
$Gag_{209-217}$-A	AAEWDRAHP	A	9
$Gag_{209-217}$-I	AAEWDRIHP	I	9
$Gag_{209-217}$-M	AAEWDRMHP	M	9
$Gag_{209-217}$-Q	AAEWDRQHP	Q	9
$Gag_{209-217}$-S	AAEWDRSHP	S	9
$Gag_{209-217}$-T	AAEWDRTHP	T	9
$Gag_{209-217}$-V	AAEWDRVHP	V	9
$Gag_{210-218}$-L	AEWDRLHPV	-	9
$Gag_{210-218}$-A	AEWDRAHPV	A	9
$Gag_{210-218}$-I	AEWDRIHPV	I	9
$Gag_{210-218}$-M	AEWDRMHPV	M	9
$Gag_{210-218}$-Q	AEWDRQHPV	Q	9
$Gag_{210-218}$-S	AEWDRSHPV	S	9
$Gag_{210-218}$-T	AEWDRTHPV	T	9
$Gag_{210-218}$-V	AEWDRVHPV	V	9
